# Comparison of Estimation Procedures for Multilevel AR(1) Models

**DOI:** 10.3389/fpsyg.2016.00486

**Published:** 2016-04-07

**Authors:** Tanja Krone, Casper J. Albers, Marieke E. Timmerman

**Affiliations:** Department of Psychometrics and Statistics, Heymans Institute, University of GroningenGroningen, Netherlands

**Keywords:** time series analysis, autocorrelation, Bayesian MCMC, multisubject, maximum likelihood estimation, simulation study

## Abstract

To estimate a time series model for multiple individuals, a multilevel model may be used. In this paper we compare two estimation methods for the autocorrelation in Multilevel AR(1) models, namely Maximum Likelihood Estimation (MLE) and Bayesian Markov Chain Monte Carlo. Furthermore, we examine the difference between modeling fixed and random individual parameters. To this end, we perform a simulation study with a fully crossed design, in which we vary the length of the time series (10 or 25), the number of individuals per sample (10 or 25), the mean of the autocorrelation (−0.6 to 0.6 inclusive, in steps of 0.3) and the standard deviation of the autocorrelation (0.25 or 0.40). We found that the random estimators of the population autocorrelation show less bias and higher power, compared to the fixed estimators. As expected, the random estimators profit strongly from a higher number of individuals, while this effect is small for the fixed estimators. The fixed estimators profit slightly more from a higher number of time points than the random estimators. When possible, random estimation is preferred to fixed estimation. The difference between MLE and Bayesian estimation is nearly negligible. The Bayesian estimation shows a smaller bias, but MLE shows a smaller variability (i.e., standard deviation of the parameter estimates). Finally, better results are found for a higher number of individuals and time points, and for a lower individual variability of the autocorrelation. The effect of the size of the autocorrelation differs between outcome measures.

## 1. Introduction

The electronic revolution allows for new and exciting research possibilities. One such possibility that has become increasingly easy to use is ecological momentary assessment (c.f., Shiffman et al., 2008; Bos et al., 2015) through electronic devices such as the mobile phone. This advancement allows, with little hassle for the individuals, multiple measurements per individual per day at the researcher's discretion (Bolger and Laurenceau, 2013). The data provided through ecological momentary assessment, often denoted as intensive longitudinal data (Hamaker et al., 2015), give ample opportunities for studying complex processes, involving the trends and dynamics of human behavior and experience. The latter pertains to studying how aspects of behavior and/or experience evolve across time, and how aspects mutually influence each other. Using these kinds of data, studies have been done pertaining to, for example, emotional complexity and age (Brose et al., 2015), dynamics of depression (Kuppens et al., 2010; Erbas et al., 2014; Kashdan and Farmer, 2014), and the relation between affect and stress (Scott et al., 2014).

Intensive longitudinal data of several individuals fall under the category of multilevel data. Multilevel data are collected according to a nested sampling design, resulting in data with a hierarchical structure (e.g., Snijders and Bosker, 1999; Hox, 2010). A two-level example is univariate longitudinal data of multiple individuals, where the time points at level 1 are nested within the individuals at level 2. In psychological sciences, momentary assessment data pertain to longitudinal series of limited length, collected among a limited number of individuals, creating a multilevel data set. To analyze these data, one can use multilevel models. In the analysis of longitudinal data, we can discern two different focuses: the trend and the dynamics across time. To study the trend across time, a multilevel regression model for repeated measures can be used. Herewith, one could use either dummy-variables (also known as indicator or design variables), to indicate effects pertaining to each time point, or time itself as a predictor (e.g., Snijders and Bosker, 1999).

To study the dynamics across time, a model is needed for describing the relationships between scores at successive measurements. This is typically done using an autoregressive model (Box and Jenkins, 1976). The simplest variant is an autoregressive model of the first order, the AR(1) model for short. For multilevel data with multiple individuals, Suls et al. (1998) used an autoregressive component in a multilevel model with random coefficients to assess change in mood over time. In this model, the autoregressive parameter was composed of a population parameter, a parameter dependent on the predictor neuroticism, and a subject dependent noise parameter. The same approach was used by Kuppens et al. (2010), who included self-esteem as a predictor for the autocorrelation. Both authors interpreted the autoregressive parameter as reflecting the degree of inertia, which is the tendency to retain the status-quo over time. An often encountered problem in time series analysis is the violation of the assumption of independent errors, due to autocorrelated noise. To account for this effect, a multilevel model including autocorrelated noise was proposed by Goldstein et al. (1994). Note that although Goldstein et al. (1994) denote this model as an autoregressive model, it is actually a moving average model, according to the common terminology (Box and Jenkins, 1976).

At the moment, it is unclear how efficient the estimation methods of different multilevel model variants are for intensive longitudinal data. Several simulation studies have been conducted to compare the different estimators for single case AR(1) models (Huitema and McKean, 1991; DeCarlo and Tryon, 1993; Arnau and Bono, 2001; Solanas et al., 2010; Krone et al., 2015). While the empirical standard error is lowest for the classical estimation method denoted by *r*_1_ (Walker, 1931), the bias is lowest for iterative estimators (Krone et al., 2015). For all methods, the empirical power is low for series with less than 50 time points. For a true autocorrelation below |0.40|, the power is below 80% for all compared estimation methods (Krone et al., 2015). This is consistent with the advice of a lower bound of 50 time points for any time series modeled with an AR(1) model, as given by Box and Jenkins (1976).

In this paper, we focus on the AR(1) model in a multilevel setting, for relatively short time series and numbers of individuals. We do so because these characteristics are typical for intensive longitudinal data, and the properties of multilevel AR(1) model estimators have been investigated scarcely. Furthermore, the inclusion of multiple individuals may have a profound effect on the bias, variability and power of the estimators. In a recent paper, Jongerling et al. (2015) compared the MLE and the Bayesian multilevel AR(1) estimators. Their simulation design included manipulations of the intercept variance and of the covariance between the autocorrelation and the error variance. However, their design lacked manipulations of the mean and variance of the autocorrelation, central to the current paper. Further, they only used person centering in MLE models and only used a random effect for the error variance in the Bayesian model, which means that their design was not fully-crossed. Jongerling et al. (2015) concluded that the estimation may be improved by including a random effect for the error variance and by refraining from person-centering. The differences in bias they found are small and inconsistent; in certain conditions increasing sample size and time series length also seems to increase rather than decrease the bias. As such, their model estimates may be biased. While they raise an interesting point with regard to individual error variances and person-centering the data, we will first consider a more basic comparison between estimation methods using the same model.

For multilevel models, several of the estimation methods used in single subject designs are unavailable. Two closed form estimators that can be used for multilevel models are generalized least squares (GLS) and generalized estimation equations (GEE) (Liang and Zeger, 1986). Although these methods have the benefit of being faster than iterative methods, i.e., MLE and Bayesian MCMC, the resulting estimates show bias and need a large amount of data points to achieve an acceptable standard error (Hox, 2010). Better fitting estimators for the ML-AR(1) model are iterative estimators, specifically the Maximum Likelihood Estimation (MLE) and the Bayesian MCMC estimation (Hox, 2010). In an earlier study, we also found this for single subject data, which leads us to use MLE and Bayesian MCMC in this paper (Krone et al., 2015).

In this paper we use a simulation study to quantify the differences between two model variants for multilevel autocorrelated data, and between two estimation methods, being MLE and Bayesian Markov-Chain Monte Carlo (Bayesian MCMC) estimation. In the next part of this paper, we discuss the multilevel model and the estimation methods. This is followed by an explanation of the simulation study design, the results of the simulation study, and a discussion on the implications for designing empirical studies involving intensive longitudinal data and properly modeling the resulting data.

## 2. The multilevel autoregressive lag 1 model

The ML-AR(1) model we use is a random coefficients model (e.g., Snijders and Bosker, 1999; Hox, 2010). The model has two levels: the first level holds the time points, as the second level holds the individuals. The level 1 model is based on the AR(1) model for a single individual (Box and Jenkins, 1976):

(1) $$\begin{array}{rc} y_{t,n}=\mu_{n}+\phi_{n}\left(y_{t-1,n}-\mu_{n}\right)+e_{t,\;n}, & \;e_{t,\;n}\sim N\left(0,\sigma_{e}\right), \end{array}$$

where *y*_*t, n*_ is the score of individual *n* (*n* = 1, 2, …, *N*) at time *t* (*t* = 1, 2, …, *T*), μ_*n*_ the intercept, ϕ_*n*_ the autocorrelation, and *e*_*t, n*_ is the error term. The error terms follow a normal distribution with mean zero and standard deviation σ_*e*_ and are independent of each other and of the observations *y*_*t, n*_.

In this paper we compare two ways of modeling multilevel data: the random model and the fixed model. The difference between these models is based on the theory behind the sampling of individuals, and is expressed in the level 2 model. In the random model, as used in the random coefficients approach, the individuals are assumed to be drawn randomly from a certain population. As such, the parameters of the individuals are assumed to be drawn randomly from the population distribution of the parameter concerned. It is common, but not required, to assume a normal distribution for the individual parameters. We will use the normality assumption in this paper.

The fixed model makes no assumption with regard to the sampling of the individuals. To reflect this, the parameters of the fixed model are estimated freely. This implicitly defines the level 2 model, as the joint distribution of the individually estimated parameters for all individuals is hereby defined. Due to the free parameter estimation, these model estimates would be the same as when the time series of each individual were modeled separately. This implies that the standard deviation of the error is σ_*e, n*_, and hence may vary across individuals.

For the random model, a level 2 model must be defined which captures the assumed population distributions of the parameters. The level 2 model we use is:

(2) $$\begin{array}{r} \mu_{n}=\gamma_{0,0}+U_{0,\;n}, \end{array}$$

(3) $$\begin{array}{r} \phi_{n}=\gamma_{0,1}+U_{1,\;n}, \end{array}$$

with:

(4) $$\begin{array}{r} U_{0,\;n}\sim N\left(0,\sigma_{U_{0,\;n}}\right), \end{array}$$

(5) $$\begin{array}{r} U_{1,\;n}\sim N\left(0,\sigma_{U_{1,\;n}}\right). \end{array}$$

where γ_0, 0_ is the population intercept, *U*_0, *n*_ is the individual specific deviation from the population intercept for individual *n*, γ_0, 1_ is the population autocorrelation and *U*_1, *n*_ is the individual specific deviation from the population autocorrelation. Note that the standard deviation of the error, σ_*e*_, is assumed to be equal across the population of individuals (unlike the fixed model), and independent of both *U*_0, *n*_ and *U*_1, *n*_. The composite model, expressing both levels in one model, is:

(6) $$\begin{array}{l} y_{t,n}=\;\gamma_{0,0}+\gamma_{0,1}(y_{t-1,n}-\gamma_{0,0}-U_{0,\;n})+U_{0,\;n}+\\ \;U_{1,\;n}(y_{t-1,n}-\gamma_{0,0}-U_{0,\;n})+e_{t,\;n},\;e_{t,\;n}\sim N(0,\sigma_{e}). \end{array}$$

### 2.1. Estimation methods

#### 2.1.1. MLE

For MLE, the distinction can be made between full maximum likelihood (FML) and restricted maximum likelihood (RML, also known as REML). The difference lies in how the likelihood is estimated: FML includes both the regression coefficients and the variance components in the likelihood, whereas RML only includes the variance components. The regression coefficients for RML are estimated in a secondary step (Hox, 2010). In general, the FML is easier to calculate. Furthermore, the FML allows for an overall chi-square test for two models that differ in the fixed part, which the RML generally does not. However, when estimating the variance, the FML model is biased since it does not take into account the number of fixed parameters (Bryk and Raudenbush, 1992, p. 46), while the RML has asymptotically unbiased variance estimates.

For the random model using MLE (henceforth denoted as MLE-R), we will use RML (Harville, 1977) with the “Bound Optimization BY Quadratic Approximation” algorithm (Powell, 2009). The method we use estimates the random parameters under the assumptions of normality, in line with typical applications in social sciences (Hox, 2010; Goldstein, 2011). The multilevel implementation of the MLE we use is not specifically made for autocorrelation measures, and may thus produce non-stationary autocorrelation values, i.e., $|{\hat{\phi }}_{n}|>1$. The number of non-stationary results obtained will be touched upon in the results section.

For the fixed model using MLE (henceforth denoted as MLE-F), we will use the ‘Broyden-Fletcher-Goldfarb-Shanno’ algorithm (Byrd et al., 1995). The estimation method we use is especially programmed for autocorrelation estimation and, as such, produces stationary autocorrelation estimates. For both MLE approaches, the algorithm may fail to reach convergence. The number of non-convergent results will be touched upon in the results section. Furthermore, both MLE approaches are unable to handle missing data, other than by removing the whole case from the analysis. To retain the data, an Expectation-Maximization algorithm (Dempster et al., 1977), also used in latent variable modeling, may be used. However, in this paper we will assume that the full data is available.

#### 2.1.2. Bayesian MCMC

Estimation through Bayesian MCMC is very versatile with respect to the models and distributions that can be estimated. The MCMC-method we use for both the fixed and random (denoted as BAY-F and BAY-R, respectively) Bayesian estimators is Hamiltonian Monte Carlo (HMC), a generalization of the Metropolis-Hastings algorithm (Metropolis et al., 1953; Hastings, 1970) that allows for an efficient estimation of the parameters (Gelman et al., 2013). An added advantage of the Bayesian approach is the possibility to deal with missing data optimally, i.e., without casewise deletion. For AR(1) models it is possible to apply the autoregressive model on the estimated score of the missing time point, instead of on the observed score itself. This allows the estimation to continue past the missing data points, adjusting the estimation as soon as the next time point is observed again.

### 2.2. Procedure

In this study, we aim to examine the comparative quality of MLE and Bayesian MCMC estimation for the autocorrelation parameter in random and fixed ML-AR(1) models. This results in four estimators which will be compared: MLE-F, MLE-R, BAY-F, and BAY-R. For the Bayesian MCMC estimations, we use the program Rstan (Stan Development Team, 2014). For the estimation of the MLE-R, we use the package lme4 for R (Bates et al., 2015). All other analyses, including data generation, are done using the functions available in the base installation of the program R (R Core Team, 2015).

## 3. Simulation study

### 3.1. Simulation design

**Figure 1:**
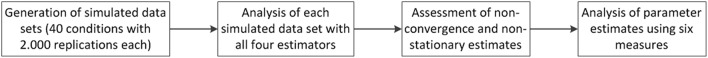
**Flowchart of the study design of the simulation study**.

To compare the four estimators for the autocorrelation, we set up a simulation design with data generation, data analyses, assessment of computational issues and analyses of the results as shown in Figure 1, with 40 conditions in total. The conditions stem from a fully crossed experimental design, including the following factors, with number of factor levels between parentheses: the length of the time series *T* (2), the number of individuals per dataset *N* (2), the standard deviation σ_*U*_1, *n*__ (2), and mean γ_0, 1_ (5) of the autocorrelation distribution, as used in Equations (5) and (3). Both *T* and *N* are either 10 or 25, σ_*U*_1, *n*__ is either 0.25 or 0.40, γ_0, 1_ is set from −0.60 up to 0.60 inclusive, taking steps of 0.30 for the values in between.

The time series were generated according to Equation (6). The mean and standard deviation of the error of each series in each replication is set to zero and one, respectively. The values of ϕ_*n*_ were then drawn from a truncated and rescaled normal distribution with range −1 to 1, to ensure the resulting time series were stationary:

(7) $$\begin{array}{r} \phi_{n}\propto N\left(\gamma_{0,1},\sigma_{U_{1,\;n}}\right)\tau \left(-1,1\right). \end{array}$$

#### 3.1.1. Parameter priors

We performed a small simulation study to examine the sensitivity for the choice of the hyperparameters of the priors of our Bayesian model. We considered 2000 replications of a single simulation condition, using 5000 iterations, taking γ_0, 0_ = 0.00, σ_*U*_1, *n*__ = 0.40, *T* = 10 and *N* = 10 (see Equation 6). This condition is one where the prior is expected to have the most influence, due to the high variability across individuals and the small amount of data. The prior we use for ${\hat{\gamma }}_{0,1}$ for BAY-R and ${\hat{\phi }}_{n}$ for BAY-F is Berger's symmetrized reference prior (Berger and Yang, 1994), which has shown to better perform than the flat prior for single case AR(1) models (Krone et al., 2015). This prior does not need hyperparameters.

We tested several hyperparameters for the prior distributions of μ_*n*_ and σ_*e*_ for the fixed model, and γ_0, 0_, σ_*U*_0, *n*__, σ_*e*_ and σ_*U*_1, *n*__ of the random model, as shown in Table 1. Our parameter of primary interest, ${\hat{\gamma }}_{0,1}$, showed small differences across the various tests. For the random model, the estimates ranged from 0.017 (test 5) to 0.026 (test 9). For the fixed model, the estimates ranged from 0.033 (test 6) to 0.109 (test 4).

**Table 1:** **Different combinations of priors tested to see their influence on the posterior results**.

**Test**	**Fixed model**		**Random model**			
	***μ_n_***	***σ_e_***	***μ***	***σ_μ_***	***σ_e_***	***σ_ϕ_***
1	*N* ~ (0, 2)	Γ ~ (2, 2)	*N* ~ (0, 2)	Γ ~ (2, 2)	Γ ~ (2, 2)	Γ ~ (2, 2)
2	*N* ~ (0, 5)	Γ ~ (2, 2)	*N* ~ (0, 5)	Γ ~ (2, 2)	Γ ~ (2, 2)	Γ ~ (2, 2)
3	*N* ~ (1, 2)	Γ ~ (2, 2)	*N* ~ (1, 2)	Γ ~ (2, 2)	Γ ~ (2, 2)	Γ ~ (2, 2)
4	*N* ~ (1, 5)	Γ ~ (2, 2)	*N* ~ (0, 2)	Γ ~ (1, 1)	Γ ~ (2, 2)	Γ ~ (2, 2)
5	*N* ~ (0, 2)	Γ ~ (1, 1)	*N* ~ (0, 2)	Γ ~ (1, 2)	Γ ~ (2, 2)	Γ ~ (2, 2)
6	*N* ~ (0, 2)	Γ ~ (1, 2)	*N* ~ (0, 2)	Γ ~ (2, 2)	Γ ~ (1, 1)	Γ ~ (2, 2)
7	*N* ~ (0, 2)	Γ ~ (2, 1)	*N* ~ (0, 2)	Γ ~ (2, 2)	Γ ~ (1, 2)	Γ ~ (2, 2)
8			*N* ~ (0, 2)	Γ ~ (2, 2)	Γ ~ (2, 2)	Γ ~ (1, 1)
9			*N* ~ (0, 2)	Γ ~ (2, 2)	Γ ~ (2, 2)	Γ ~ (1, 2)

For the random estimator, the estimated γ_0, 0_ showed small differences across the various priors, resulting in estimates ranging from 0.000 (test 9) to 0.004 (test 3). For the fixed estimator, the estimated ${\bar{\mu }}_{n}$ ranged from 0.000 (test 7) to 0.192 (test 2). The effect of the different priors is most notable for the posterior of the parameter for which the prior was changed. For the simulation study, we use the priors of test 1 of Table 1, as these gave the best results.

#### 3.1.2. Number of iterations

A preliminary study was performed to decide on the number of iterations needed for the Bayesian MCMC. Because of the more complicated model of BAY-R compared to BAY-F, we only tested the number of iterations for BAY-R. Ten datasets per condition were used to find the convergence rate as expressed through the potential scale reduction factor $\hat{R}$, as can be seen in Table 2. The potential scale reduction factor shows the ratio of how much the estimation may change when the number of iterations is doubled, with a value of 1 indicating that no change is expected (Gelman and Rubin, 1992; Stan Development Team, 2014). We deemed the improvements brought by a higher number of iterations negligible, thus we continued using 3000 total iterations, of which 1500 were burn-in.

**Table 2:** **Values of Ř for different amounts of iterations for tests with 10 replications per condition using the BAY-R method and for the final analyses**.

**Iterations**		**Mean**	**Percentage of Ř above:**			
**Total**	**Burn-in**	**Ř**	**1.05**	**1.1**	**1.5**	**1.7**
3000	1500	1.01	2.53	0.89	0.02	0.00
4000	2000	1.01	1.97	0.68	0.02	0.00
10,000	5000	1.00	1.54	0.75	0.01	0.00
10,000	8000	1.01	2.13	0.70	0.01	0.00
**Final analyses: 3000 iterations with 1500 burn-in**						
BAY-R		1.00	0.94	0.35	0.01	0.00
BAY-F		1.00	0.02	0.00	0.00	0.00

#### 3.1.3. Number of replications

A preliminary study using *N* = 10, *T* = 10, σ_*U*_1, *n*__ = 0.40, and γ_0, 1_ = −0.30, with the priors and number of iterations as specified, showed that the outcome measures (to be introduced in the next section) started stabilizing after around 1500 replications for all used methods, being stable for all at 2000 replications. For example, the standard deviations of the estimated mean γ_0, 1_ or ϕ_*n*_, depending on estimation method, over replications was lower than 0.01 at 2000 replications for all used estimators. Therefore, the number of replications per condition is set to *R* = 2000. Given that we have 40 conditions, this amounts to 40 × 2000 = 80, 000 datasets generated.

#### 3.1.4. Summary

Using this simulation design, we can define our study using the classification for intensive longitudinal data designs as discussed by Hamaker et al. (2015). We analyze multi-subject data (where the single-subject case can be seen as a special case). Since we use the classic AR(1) model, we model a univariate, stationary, linear process in discrete time. Our variable has a continuous distribution and is based in the time-domain. Finally, we model the process and are primarily interested in the parameters characterizing the process, rather than the descriptive statistics.

In our simulation study, we consider two measures of computational problems (i.e., non-convergence and non-stationary estimates), and six different outcome measures for the autocorrelation: the bias of ${\hat{\gamma }}_{0,1}$, the bias of ${\hat{\sigma }}_{U_{1,\;n}}$, the empirical standard deviation of ${\hat{\gamma }}_{0,1}$, the bias of the standard error of ${\hat{\gamma }}_{0,1}$, the empirical rejection rate (EPr) of ${\hat{\gamma }}_{0,1}$ and the point and interval estimates of ${\hat{\gamma }}_{0,1}$. For each outcome measure, we offer a short explanation of the measurement and the obtained results.

### 3.2. Results

We start with discussing the rates of non-convergence (MLE-F) and non-stationarity (MLE-R), followed by the outcome measures for the autocorrelation. We will only discuss the conditions where an effect was found; thus if the random estimator is named but not the fixed estimator, the condition discussed does not influence the result of the fixed estimator and vice versa. The graphs presented in this section show the outcome measures as a function of *N*, *T*, σ_*U*_1, *n*__ and γ_0, 1_. The model parameters will be discussed in the notation used in Equation (6), the statistics obtained with the random and fixed estimators in their respective notations as in Equations (6) and (1).

#### 3.2.1. Computational problems: non-convergence and non-stationary estimates

The MLE-F is occasionally unable to reach convergence in the estimation of the model, which is connected to the inability to estimate values outside the range of −1 to 1. Of the 40 conditions, 28 converged for all analyses performed. In total, 0.002% of the estimates did not reach convergence. The highest percentage of non-convergence for individual time series is 0.01% for the condition with *N* = 10, *T* = 25, σ_*U*_1, *n*__ = 0.25, and γ_0, 1_ = 0.6. Apart from the condition with the highest number of non-convergence, higher numbers of non-convergence are found for conditions with larger values of |ϕ| and conditions with the highest value of σ_*U*_1, *n*__.

Out of the 40 conditions, only three had purely stationary estimates. In total 0.33% of the estimates were non-stationary. The highest percentage of non-stationary values for the MLE-R was 1.23%, for the condition with *N* = 10, *T* = 10, σ_*U*_1, *n*__ = 0.40, and γ_0, 1_ = −0.60. As expected, higher numbers of non-stationary estimates were found for higher values of |γ_0, 1_| and for the highest value of σ_*U*_1, *n*__.

Thus, although we found non-convergence and non-stationarity in some cases, their low occurrence indicate that the problems caused by these issues are minor.

#### 3.2.2. Bias of ${\hat{\gamma }}_{0,1}$

The bias of the ${\hat{\gamma }}_{0,1}$ indicates whether a systematic under- or overestimation of γ_0, 1_ is found. The bias is computed as:

(8) $$\begin{array}{r} \text{bias}=\left(\frac{1}{R}\overset{R}{\underset{r=1}{\sum }}{\hat{\gamma }}_{0,1_{r}}\right)-\gamma_{0,1}, \end{array}$$

where *r*(*r* = 1, 2, …, *R*) refers to the replication number. The random estimators estimate ${\hat{\gamma }}_{0,1}$ directly. For the fixed estimators, ${\hat{\gamma }}_{0,1}$ is estimated as $\frac{1}{N}\overset{N}{\underset{n=1}{\sum }}{\hat{\phi }}_{n}$.

**Figure 2:**
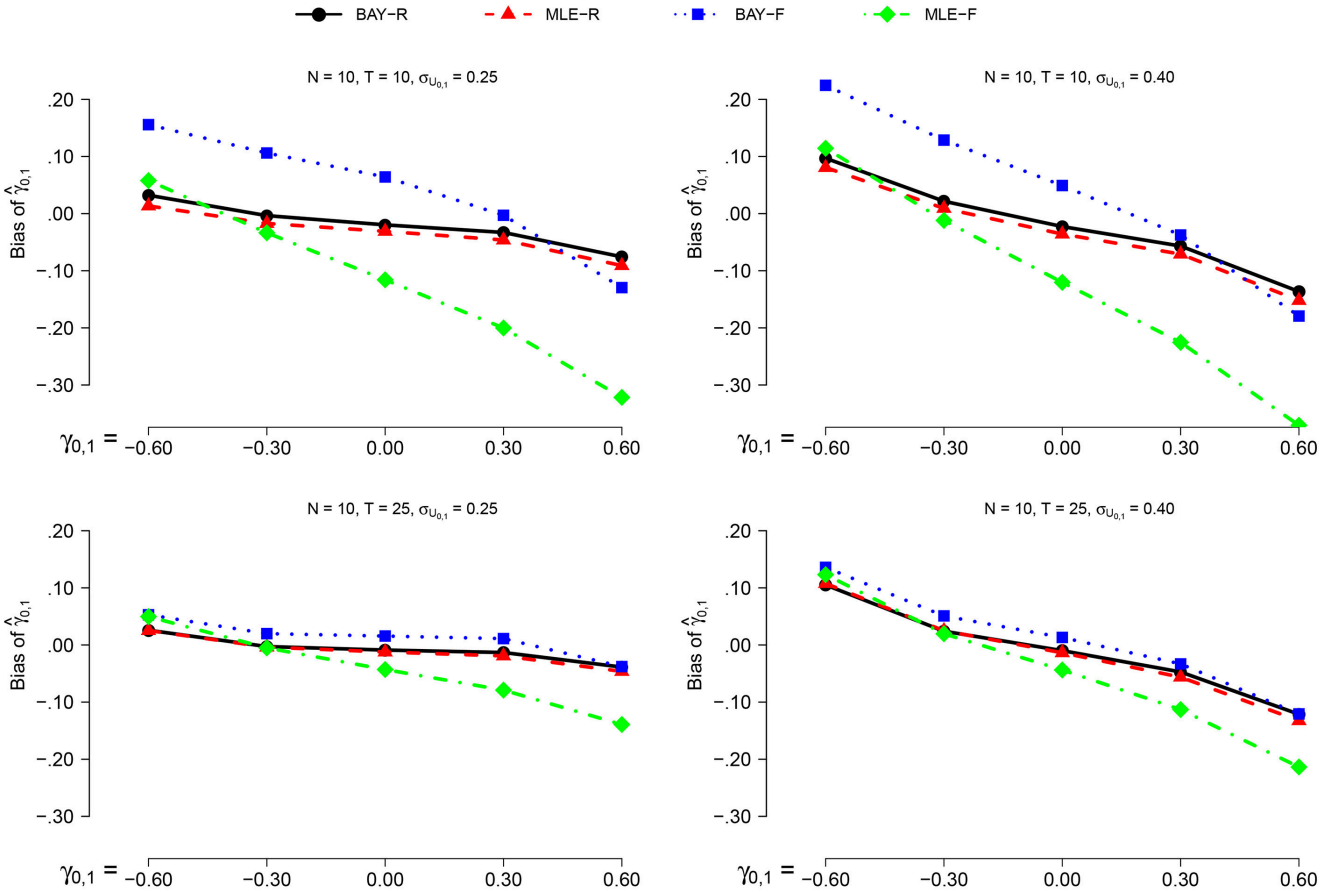
**The bias of ${\hat{\gamma }}_{0,1}$ for *N* = 10 for the different estimators, different time series length *T* and different values of σ_γ_0, 1__ as a function of γ_0, 1_**.

The bias decreases marginally for *N* = 25 compared to *N* = 10, with the largest difference being −0.05 for MLE-R, in the conditions with *T* = 25, σ_*U*_1, *n*__ = 0.25 and γ_0, 1_ = 0.6. This prompted us to only show the results for *N* = 10, see Figure 2. The bias decreases for *T* = 25 compared to *T* = 10 for the fixed methods. For σ_*U*_1, *n*__ = 0.25 compared to σ_*U*_1, *n*__ = 0.40, the bias decreases for all methods. A trend is present, where the value of the bias of ${\hat{\gamma }}_{0,1}$ decreases as γ_0, 1_ increases. The bias is, in general, positive for negative values of γ_0, 1_, and negative for positive values of γ_0, 1_.

As can be seen in Figure 2, the random estimators, BAY-R and MLE-R, show a smaller bias than the fixed estimators, MLE-F and BAY-F. This difference is larger when *T* = 10 compared to *T* = 25. The difference between MLE-R and BAY-R is very small and inconsistent over conditions. For γ_0, 1_ above 0.00, the bias of MLE-F is larger than the bias of BAY-F; for γ_0, 1_ below 0.00, this is the other way around.

#### 3.2.3. Bias of ${\hat{\sigma }}_{U_{1,\;n}}$

The bias of ${\hat{\sigma }}_{U_{1,\;n}}$ indicates whether ${\hat{\sigma }}_{U_{1,\;n}}$ is systematically under- or overestimated, and is calculated as:

(9) $$\begin{array}{r} \text{bias}=\left(\frac{1}{R}\overset{R}{\underset{r=1}{\sum }}{\hat{\sigma }}_{U_{1,\;n}}\right)-\sigma_{U_{1,\;n}}. \end{array}$$

The random estimators estimate ${\hat{\sigma }}_{U_{1,\;n}}$. For the fixed estimators, ${\hat{\sigma }}_{U_{1,\;n}}$ is calculated per replication *r* as $SD\left({\hat{\phi }}_{n}\right)$.

**Figure 3:**
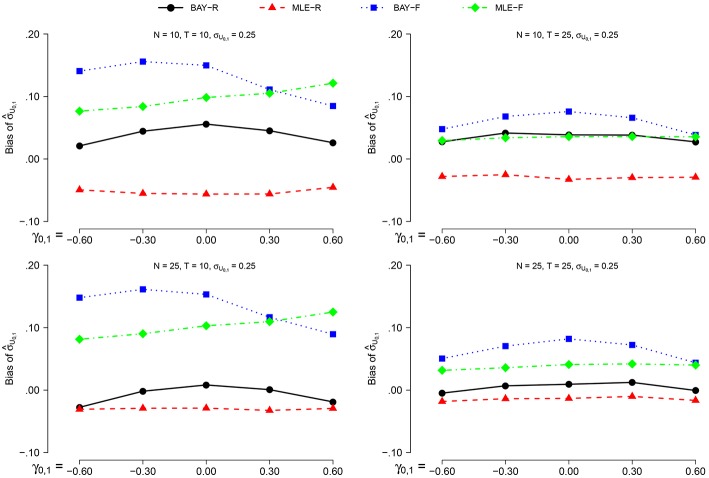
**The bias of ${\hat{\sigma }}_{U_{1,\;n}}$ for σ_ϕ_*n*__ = 0.25 for the different estimators and different group sizes *N* for timeseries of different length *T* as a function of γ_0, 1_**.

The bias of ${\hat{\sigma }}_{U_{1,\;n}}$ is smaller for σ_*U*_1, *n*__ = 0.40 than for σ_*U*_1, *n*__ = 0.25 for all estimators. As the pattern over the other conditions stays the same, we only show the results for σ_*U*_1, *n*__ = 0.25, as depicted in Figure 3. For the random estimators, the bias for *N* = 25 is smaller than the bias for *N* = 10. The bias is smaller for *T* = 25 than for *T* = 10, with a more pronounced effect for the fixed estimators. The effect of γ_0, 1_ is small and inconsistent between conditions and estimators.

BAY-R shows the lowest bias, followed by MLE-R, except for the combination of σ_*U*_1, *n*__ = 0.40, *N* = 10 and *T* = 25, where MLE-F shows a smaller bias than both MLE-R and BAY-R. For all conditions, the bias of ${\hat{\sigma }}_{U_{1,\;n}}$ is largest for BAY-F.

#### 3.2.4. Empirical $SD\left({\hat{\gamma }}_{0,1}\right)$

The empirical, or observed, standard deviation ($SD\left({\hat{\gamma }}_{0,1}\right)$) indicates the variability of ${\hat{\gamma }}_{0,1}$. The empirical SD is computed as the standard deviation of ${\hat{\gamma }}_{0,1}$ over the *R* replications for the random estimators, and as the standard deviation of $\frac{1}{N}\overset{N}{\underset{n=1}{\sum }}{\hat{\phi }}_{n}$ over replications for the fixed estimators.

**Figure 4:**
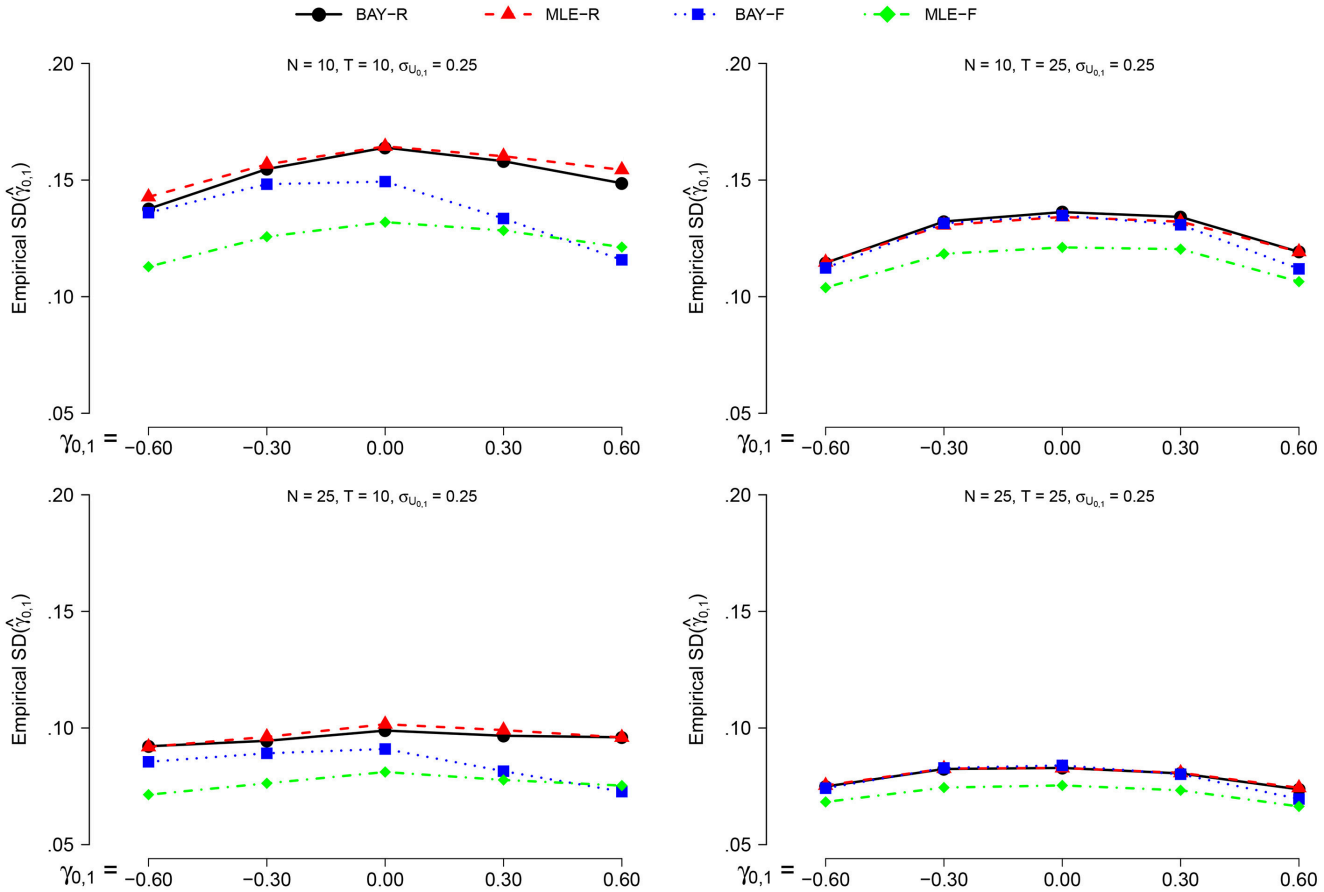
**The empirical $SD\left({\hat{\gamma }}_{0,1}\right)$ for σ_ϕ_*n*__ = 040 for the different estimators and different group sizes *N* for timeseries of different length *T* as a function of γ_0, 1_**.

The empirical $SD\left({\hat{\gamma }}_{0,1}\right)$ is larger for σ_*U*_1, *n*__ = 0.40 than for σ_*U*_1, *n*__ = 0.25, on average by a factor of 1.2. The effect of all other parameters is equal for both values of σ_*U*_1, *n*__, prompting us to only display the $SD\left({\hat{\gamma }}_{0,1}\right)$ for σ_*U*_1, *n*__ = 0.40, as can be seen in Figure 4. The $SD\left({\hat{\gamma }}_{0,1}\right)$ is smaller for *N* = 25 compared to *N* = 10, and for *T* = 25 compared to *T* = 10. Extreme values of γ_0, 1_ give a lower $SD\left({\hat{\gamma }}_{0,1}\right)$, but only marginally.

The random estimators show a larger $SD\left({\hat{\gamma }}_{0,1}\right)$ than the fixed estimators. The smallest empirical SD is shown by the MLE-F, followed by the BAY-F. The difference between the MLE-R and BAY-R is small and practically negligible.

#### 3.2.5. Bias of $SE\left({\hat{\gamma }}_{0,1}\right)$

The bias of the standard error indicates how well the methods estimate the standard deviation of ${\hat{\gamma }}_{0,1}$. The bias of $SE\left({\hat{\gamma }}_{0,1}\right)$ is calculated as:

(10) $$\text{bias of SE }({\hat{\gamma }}_{0,1})=\left(\frac{1}{\text{R}}\sum_{r\;=\;1}^{R}SE({\hat{\gamma }}_{0,1_{r}})\right)-SD({\hat{\gamma }}_{0,1}),$$

where $SE\left({\hat{\gamma }}_{0,1_{r}}\right)$ is the standard error of ${\hat{\gamma }}_{0,1}$ in replication *r*. For the random estimators, the $SE\left({\hat{\gamma }}_{0,1_{r}}\right)$ is the standard error as calculated by the estimator. For the fixed estimators, the SE is taken as $\frac{1}{N}\overset{N}{\underset{n=1}{\sum }}SE\left({\hat{\phi }}_{n}\right)$.

**Figure 5:**
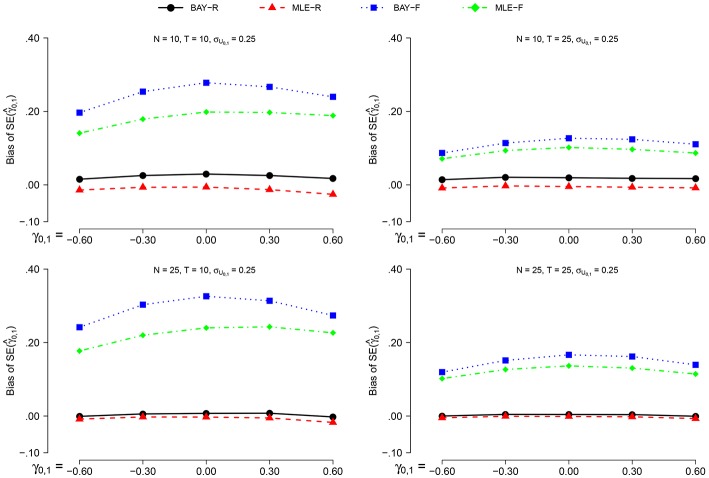
**The bias of $SE\left({\hat{\gamma }}_{0,1}\right)$ for σ_ϕ_*n*__ = 0.25 for the different estimators and different group sizes *N* for timeseries of different length *T* as a function of γ_0, 1_**.

The bias of $SE\left({\hat{\gamma }}_{0,1}\right)$ is smaller when σ_*U*_1, *n*__ = 0.40 than when σ_*U*_1, *n*__ = 0.25. However, the effect of all other parameters on the bias of $SE\left({\hat{\gamma }}_{0,1}\right)$ is equal for both values of σ_*U*_1, *n*__, prompting us to display the results for σ_*U*_1, *n*__ = 0.25 only, as can be seen in Figure 5. For the random estimators, *N* = 25 gives a smaller bias than *N* = 10, for the fixed estimators this is the other way around. The effect of *T* is only present for the fixed estimators, which show a smaller bias of $SE\left({\hat{\gamma }}_{0,1}\right)$ for *T* = 25 than for *T* = 10. For the fixed estimators, this effect is stronger than the effect of *N*. The different values of γ_0, 1_ only influence the estimations of the fixed estimators, which show a slightly smaller bias for higher values of |γ_0, 1_|.

The MLE-R shows the smallest bias of $SE\left({\hat{\gamma }}_{0,1}\right)$ for all conditions, and is the only estimator which shows a constant negative bias. For higher values of *N*, the difference between MLE-R and BAY-R disappears. For all conditions, the bias of $SE\left({\hat{\gamma }}_{0,1}\right)$ is larger for the fixed estimators than for the random estimators.

#### 3.2.6. Empirical rejection rate and power

For each estimator and condition, we compute the empirical probability (EPr) for rejecting *H*_0_:γ_0, 1_ = 0 in favor of *H*_α_:γ_0, 1_ ≠ 0.00, with α = 0.05. Using frequentist terminology, the EPr equals the actual α in the condition with γ_0, 1_ = 0.00; and the power in all other conditions.

For frequentist methods, testing *H*_0_:γ_0, 1_ = 0 vs. a two-sided alternative at significance level α, is equivalent to checking whether the (1−α) confidence interval (CI) includes zero or not. The CI per replication per condition and per estimator is calculated as follows:

(11) $$\begin{array}{r} {\hat{\gamma }}_{0,1}\pm t_{\left(1-\alpha \right);df=\;N-2}^{*}SE\left({\hat{\gamma }}_{0,1}\right), \end{array}$$

where $SE\left({\hat{\gamma }}_{0,1}\right)$ is obtained as explained in Section 3.2.5. The proportion of replications per condition for which the corresponding confidence interval does not contain zero is the EPr.

For the Bayesian estimators, the EPr is the proportion of replications per condition for which the credible interval (CrI) as obtained through MCMC does not hold zero. For the BAY-R, we consider the CrI of $\hat{\phi }$, for BAY-F we use the average scores of the CrI's of ${\hat{\phi }}_{n}$ within each replication.

**Figure 6:**
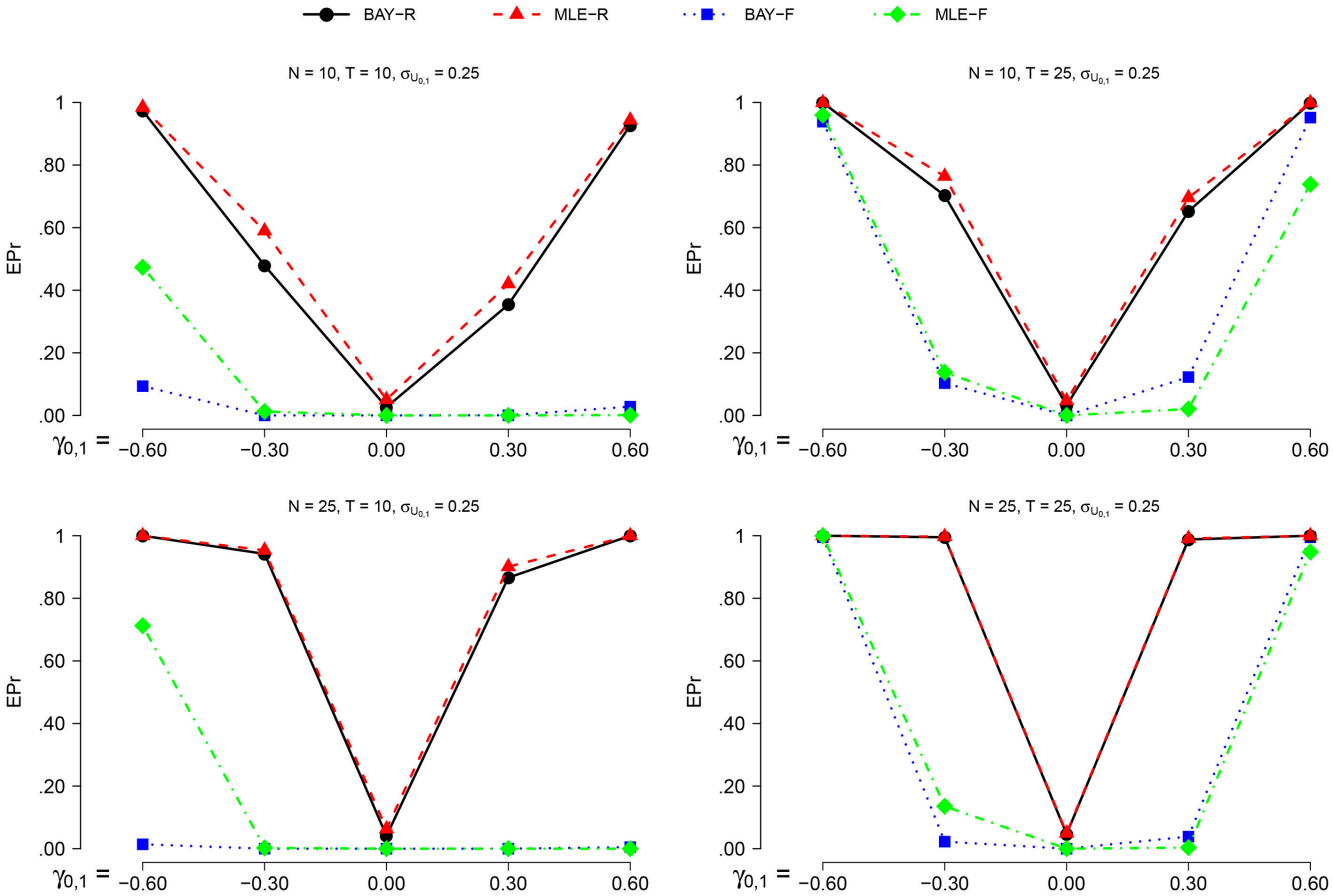
**The EPR for σ_*U*_1, *n*__ = 0.25 for the different estimators, different group sizes *N*, and for different timeseries length *T* as a function of γ_0, 1_**.

**Figure 7:**
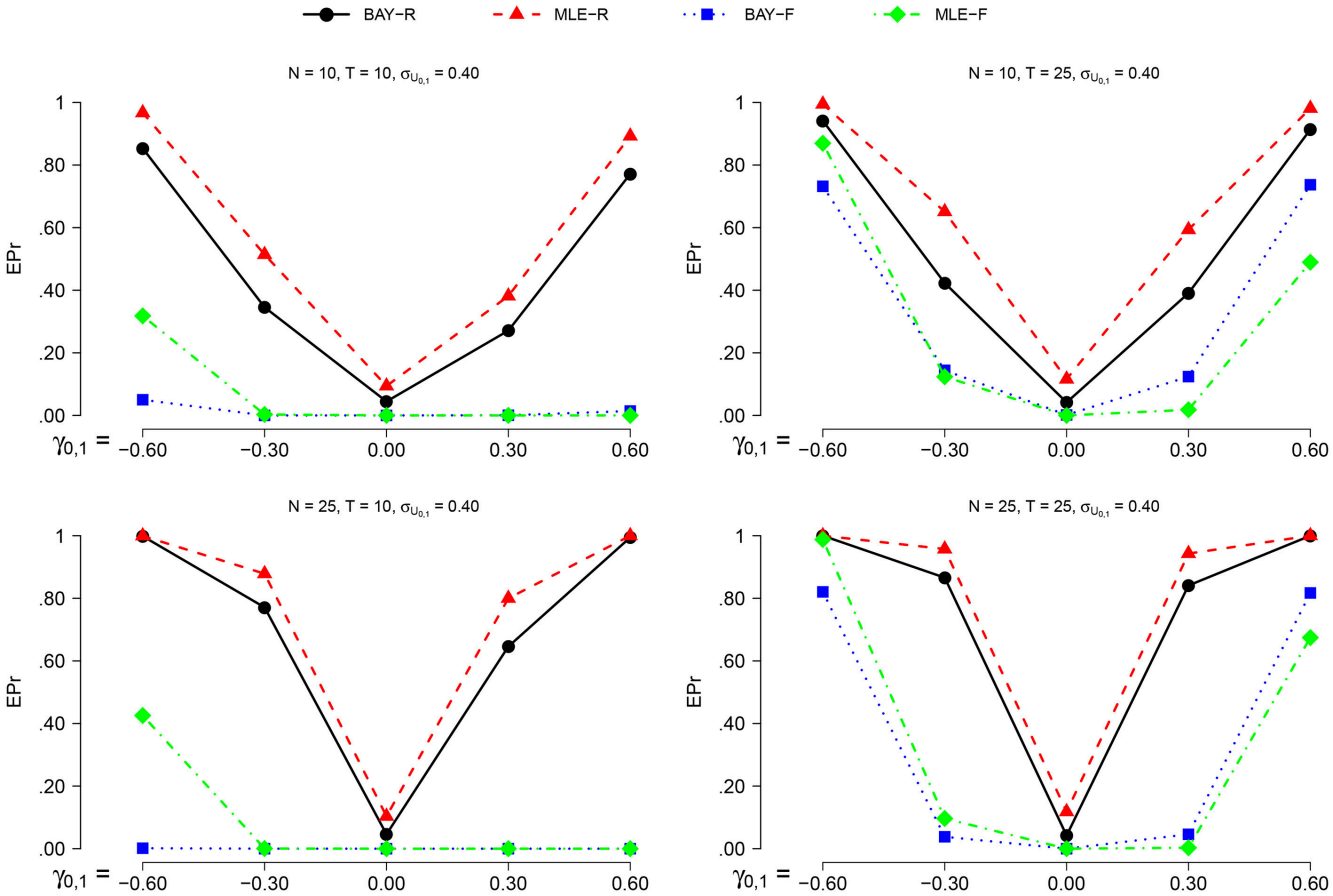
**The EPR for σ_*U*_1, *n*__ = 0.40 for the different estimators, different group sizes *N*, and for different timeseries length *T* as a function of γ_0, 1_**.

The power is higher for *N* = 25 than for *N* = 10 and for *T* = 25 compared to *T* = 10, as can be seen in Figures 6, 7. The actual α shows no such effect. The EPr shows lower values for σ_*U*_1, *n*__ = 0.40 compared to σ_*U*_1, *n*__ = 0.25, except for the actual α of MLE-R. When |γ_0, 1_| is higher, the EPr becomes higher. For the fixed estimators, this effect is strongly dependent on *T*: for *T* = 10, the EPr only increases for γ_0, 1_ < −0.30.

The highest power is found using BAY-R when σ_*U*_1, *n*__ = 0.25, and using MLE-R when σ_*U*_1, *n*__ = 0.40. For the fixed estimators, the BAY-F shows a higher power than the MLE-F. The BAY-R has an actual α consistently at or around 0.05, while the MLE-R has an actual α that is too high for σ_*U*_1, *n*__ = 0.40, namely at 0.10. The fixed estimators have an actual α at or even below 0.01, rather than the desired 0.05.

#### 3.2.7. Point and interval estimates of γ_0, 1_

To illustrate the joint effects of bias and variability we consider BAY-R and MLE-R, using the point and interval estimates of γ_0, 1_. As point estimate we use the mean of ${\hat{\gamma }}_{0,1}$ per condition. For the interval estimation we present the 2.5 and 97.5 percentiles of the ${\hat{\gamma }}_{0,1}$ across all *R* replications per condition as the lower and upper bounds.

**Figure 8:**
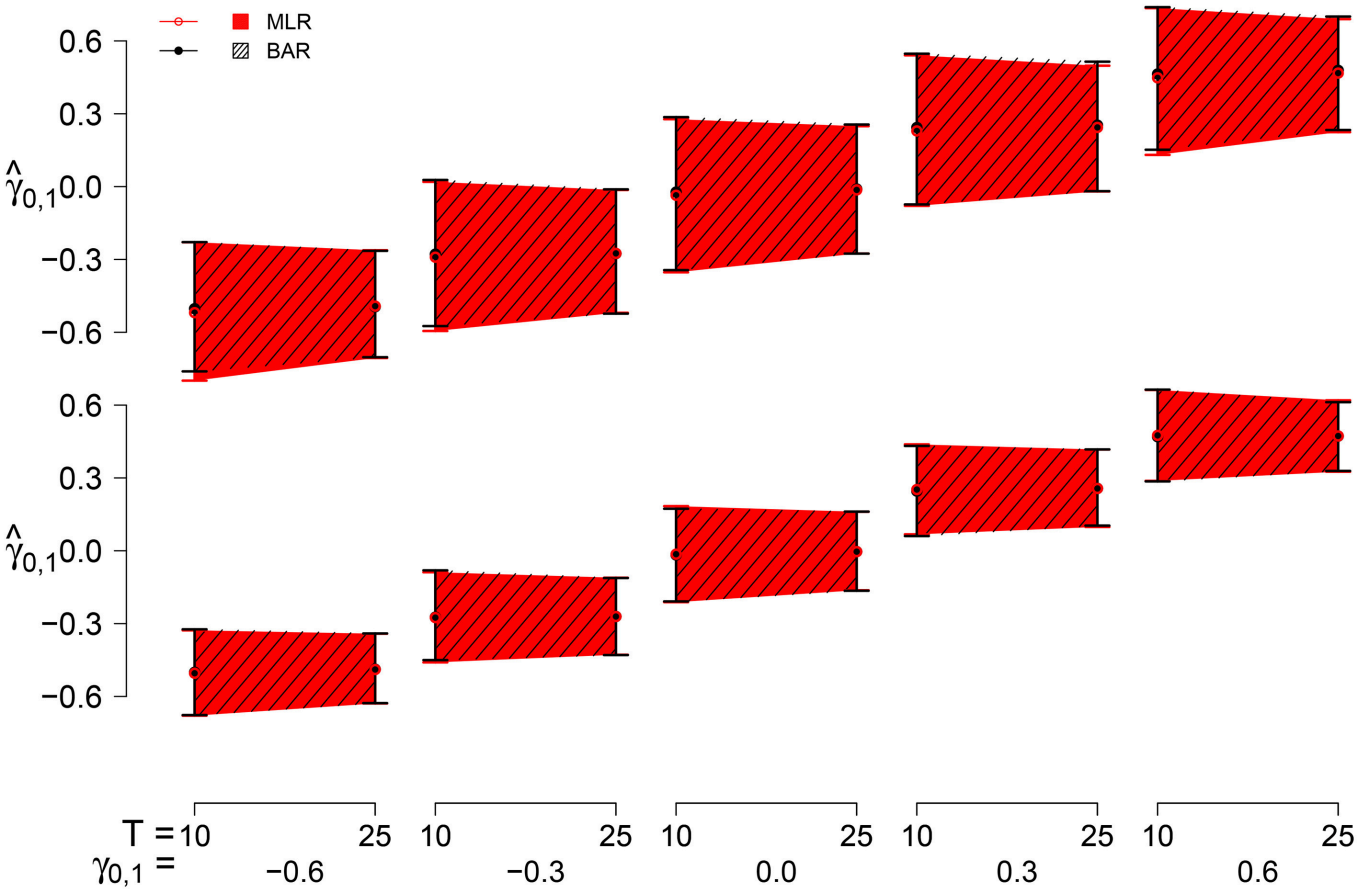
**The point and interval estimates for σ_ϕ_*n*__ = 0.40 for the different estimators and different group sizes *N* (*N* = 10 top pane, *N* = 25 bottom pane) for time series length *T* = 10 and *T* = 25 as a function of γ_0, 1_**.

The point estimates and interval estimates can be seen in Figure 8 for σ_*U*_1, *n*__ = 0.40. The interval is larger for *N* = 10 and for *T* = 10 than for *N* = 25 and *T* = 25. The effect of *N* is slightly larger. σ_*U*_1, *n*__ = 0.25 effectuates a smaller estimation interval than σ_*U*_1, *n*__ = 0.40, the latter being 1.2 to 1.3 times the former. The influence of γ_0, 1_ on the estimation interval is negligible, as are the differences between BAY-R and MLE-R.

### 3.3. Combined conclusions of the different measures

We found that the use of random estimators as opposed to fixed estimators improves all measurements considerably, except for the empirical SD, which is larger for the random estimators. The BAY-R shows a slight advantage over the MLE-R with respect to the bias of ${\hat{\sigma }}_{U_{1,\;n}}$ and the bias of $SE{\hat{\gamma }}_{0,1}$. As expected, higher values of *N* and *T* improve the estimation. Further, as expected, a lower value of σ_*U*_1, *n*__ lowers the bias of ${\hat{\gamma }}_{0,1}$, lowers the $SD\left({\hat{\gamma }}_{0,1}\right)$ and increases the power, but also increases the bias of ${\hat{\sigma }}_{U_{1,\;n}}$ and the bias of $SE\left({\hat{\gamma }}_{0,1}\right)$.

## 4. Discussion and conclusions

In this paper we studied the performance of four models for multilevel time-series data. We compared two estimation methods, namely maximum likelihood estimation and Bayesian MCMC, as previous work indicates that these methods perform best for single case designs (Krone et al., 2015). We combined this with two model variants, a random model and a fixed model, to obtain four estimators: MLE-F, MLE-R, BAY-F, and BAY-R. We compared their estimates in different conditions, where we varied the time series lengths, number of subjects and the mean and standard deviation of the autocorrelation distribution. As outcome measures, we considered the bias, the bias of the standard deviation, the empirical standard deviation, the bias of the standard error, the empirical rejection rate, and the point and interval estimates of the autocorrelation.

We found substantial differences between the fixed and the random estimators. When compared to the fixed estimators, the random estimators show better results for the bias, the bias of the standard deviation, the bias of the standard error and the power. Furthermore, the actual α as obtained with the fixed estimators, appears to be between 0.00 and 0.01, in stead of 0.05. The fixed estimators show a better empirical standard deviation than the random estimators. In general, the random estimators are clearly preferred over the fixed estimators.

Smaller differences were found between the estimation methods. In general, the Bayesian MCMC shows a smaller bias than the MLE. The bias of the standard deviation is smaller for BAY-R than for MLE-R, but smaller for MLE-F than for BAY-F. The empirical standard deviation is smaller for the MLE-F than for the BAY-F, but the difference between BAY-R and MLE-R is negligible. The bias of the standard error is smaller for the MLE. The power is higher for the MLE estimators, but the actual α is better for the Bayesian MCMC. In general, the bias of the estimated autocorrelation is smaller for the Bayesian MCMC, but the variability is smaller for the MLE estimators.

The effect of the different conditions depends on the model variant. A higher sample size *N* improves all outcome measurements for the random estimators. For the fixed estimators, a higher *N* marginally improved the bias, the empirical standard deviation and the power of the autocorrelation. However, although the increase in *N* decreased the empirical standard deviation, it did not influence the estimation of the standard error, thus increasing the bias of the standard error for *N* = 25.

The time series length *T* influences the estimations for both model variants. A higher value of *T* showed small but positive effects on the outcome measures for the random estimators. However, the improvement was smaller than for an equal increase in *N*. For the fixed estimators, the results were more profound, showing stronger improvements in all outcome measures than obtained for an equal increase in *N*.

The standard deviation of ϕ_*n*_ influenced the results for all estimators and conditions. A higher σ_*U*_1, *n*__ gave less favorable results for the autocorrelation with regard to bias, empirical standard deviation and power, but more favorable results for the bias of the standard deviation and the bias of the standard error. The effect of the mean of ϕ_*n*_, γ_0, 1_, differs per estimator and per condition, not showing a clear pattern between estimators and conditions. Earlier studies showed a negative relation between the bias and γ_0, 1_: a negative γ_0, 1_ gave a positive bias, and the other way around (e.g., Huitema and McKean, 1991; DeCarlo and Tryon, 1993; Solanas et al., 2010). This result was replicated.

An important question in time series analysis is how many individuals and time points are needed to obtain acceptable estimates for a given model. In choosing between a random or a fixed approach to modeling, the random modeling is clearly favored when the assumptions associated with the model do hold. In this case, more individuals can be used to make up for a smaller number of time points, and the other way around. When σ_*U*_1, *n*__ is up to 0.25, the random model may produce results with an acceptable size of bias when *T* or *N* is at least 25, and the other one of the two is at least 10. When σ_*U*_1, *n*__ is up to 0.40, both are required to be higher than 25. The number of individuals only has a small effect on the results for the fixed model. Here, the number of time points is the strongest criteria. In this study, we still found a sizable bias for 25 time points, which is stronger for σ_*U*_1, *n*__ = 0.40. This is confirmed in single subject studies, where a *T* of 50 is advised (Box and Jenkins, 1976; Krone et al., 2015).

The aim of this paper was to compare the four estimators MLE-F, MLE-R, BAY-F, and BAY-R using a multilevel AR(1) model. For the single subject AR(1) model, several issues and important factors are discussed in the literature. These may be just as relevant for a multisubject model, such as our multilevel model. The AR(1) model, though very often used, is not sophisticated enough for various empirical applications. This is because the error term (*e*_*t, n*_ in Equation 6) is also affected by the auto-correlation. Schuurman et al. (2015) demonstrates that including so-called white noise (i.e., error not carried over to the next time point) in the model, leads to improved empirical model fit. Lacking this term leads to underestimation of the absolute autocorrelation. Studying how various estimators perform under such an extension to the (multilevel) AR(1) model is an interesting step in future research.

The literature on the single subject AR(1) model discusses several other factors that influence the estimation of the autocorrelation. In our models we kept the error variance equal for all datasets, but this does influence the estimation of the AR(1) model (Schuurman et al., 2015), as does the error distribution (Solanas et al., 2010). This may also influence the performance of the different estimators as used in this paper. Another issue is misspecification, where the model used may not be equal to the one underlying the data. Earlier studies showed that this influences the estimation of the autocorrelation (Tanaka and Maekawa, 1984; Kunitomo and Yamamoto, 1985; Krone et al., 2015). For the multilevel model, the inclusion of a random error covariance may improve estimation, while person-centering may have a negative effect on the estimation of the parameters (Jongerling et al., 2015). The effect of these factors on the different estimators in a multilevel model is also an interesting topic for further studies.

We chose a well-known multilevel framework for our estimators, which is often used in longitudinal analyses. An alternative framework to model an AR-model is a State Space Model (SSM) (Durbin and Koopman, 2012). The versatility of the SSM means that it can be used for a vast range of models and any distribution for which a link-function with the normal distribution exists. Furthermore, the implementation of measurement error parameters is straightforward in a SSM. SSM can be modeled to allow for a multilevel AR(1) structure for different kinds of distributions; implementations have been made for normally distributed data (Lodewyckx et al., 2011) and data following a Poisson distribution (Terui et al., 2010). However, the theoretical framework to estimate a SSM with any distribution in the exponential family is available (Petris et al., 2009; Durbin and Koopman, 2012). A Bayesian interpretation of the state space model is found in the Bayesian dynamic model (West and Harrison, 1997).

We compared several estimators, but many other possibilities remain. Future studies may look into the effect of data properties, such as the error variance or misspecification, and different ways of modeling the data, using for example a SSM framework. Finally, we did not assess how the estimators handle missing data, and what the effect of missing data is on the outcome measures. As missing data occurs often in the social sciences, this is an interesting and important topic for further studies.

## Author contributions

TK: Main contributing author, CA and MT: co-authors of the study-design, substantial contributions to the manuscript and revisions thereof.

## Funding

This work is funded by the Netherlands Organization for Scientific Research (NWO), grant number 406-11-018.

### Conflict of interest statement

The authors declare that the research was conducted in the absence of any commercial or financial relationships that could be construed as a potential conflict of interest.
